# Nonsexual violence against children and adolescents: a study in a Latin American tertiary and university hospital

**DOI:** 10.1590/1984-0462/2022/40/2021101IN

**Published:** 2022-05-06

**Authors:** Reinan Tavares Campos, Lorena Vasconcelos Mesquita Martiniano, Amanda Kerlyn Santos Lirio, Kalesa Elias de Araujo Souza, Natalia Rose, Juliana Martins Monteiro Dias, Antônio Carlos Alves Cardoso, Sylvia Costa Farhat, Clovis Artur Silva

**Affiliations:** a Universidade de São Paulo, São Paulo, SP, Brazil.

**Keywords:** Violence, Chronic disease, Child, Adolescent, Emergency, Bullying, Violência, Doença crônica, Criança, Adolescente, Emergências, Bullying

## Abstract

**Objective:**

The objective of this study was to assess interpersonal nonsexual violence against children and adolescents in a tertiary university hospital.

**Methods:**

A cross-sectional study was performed in 240 patients under nonsexual violence situation for 15 consecutive years. Data analyses included demographic data, hospital referral site, type and author of nonsexual violence, legal referral, laboratorial and imaging examinations, and outcomes.

**Results:**

Nonsexual violence situation was diagnosed in 240 (0.1%) of 295,993 patients for 15 years: 148 (61.7%) in children and 92 (38.3%) in adolescents. Out of 240, the most frequent types of violence were negligence in 156 (65.0%), physical 62 (25.8%), psychological/emotional aggression 52 (21.7%), Munchausen by proxy syndrome 4 (1.7%), and bullying/cyberbullying in 3 (1.3%). Out of 123, the most common pediatric chronic conditions were chronic kidney disease 24 (19.5%), human immunodeficiency virus 14 (11.4%), prematurity 9 (7.3%), cerebral palsy 8 (6.5%), and asthma 8 (6.5%). Further comparison between children *versus* adolescent under nonsexual violence situation revealed significant difference between the hospital referral sites. The frequency of patients under violence referred from outpatient clinics was significantly reduced in children *versus* adolescents (27.7 vs. 62%), whereas emergency department was higher in the former group (57.4 vs. 25.0%; p<0.001). All types of violence situations and pediatric chronic conditions were similar in both groups (p>0.05).

**Conclusions:**

Nonsexual violence against our pediatric population was rarely diagnosed in a tertiary hospital, mainly negligence, physical, and psychological/emotional aggression. Approximately two-thirds of violence diagnosis occurred in children, referred mainly by the emergency department. In contrast, approximately one-third of violence diagnosis occurred in adolescents, referred mostly by outpatient clinics.

## Abstract (pt)

**Objetivo:**

Avaliar violência interpessoal não sexual contra crianças e adolescentes em um hospital universitário terciário.

**Métodos:**

Foi realizado um estudo transversal com 240 pacientes em situação de violência não sexual por 15 anos. A análise incluiu: dados demográficos, local de referência do hospital, tipo e autor da violência, encaminhamento legal, exames laboratoriais e de imagem, e desfechos graves.

**Resultados:**

Situações de violência não sexual foram diagnosticadas em 240 de 295.993 (0,1%) pacientes durante os 15 anos do período: 148/240 (61,7%) em crianças e 92/240 (38,3%) em adolescentes. Os tipos de violências mais frequentes foram negligência em 156/240 (65,0%), violência física 62/240 (25,8%), agressão psicológica/emocional 52/240 (21,7%), síndrome de Münchausen por procuração 4/240 (1,7%) e bullying/cyberbullying em 3/240 (1,3%). As condições crônicas pediátricas mais frequentes foram: doença renal crônica 24/123 (19,5%), vírus da imunodeficiência humana 14/123 (11,4%), prematuridade 9/123 (7,3%), paralisia cerebral 8/123 (6,5%) e asma 8/123 (6,5%). Comparações entre crianças e adolescentes em situação de violência não sexual revelaram que houve diferença significativa entre os locais de referência do hospital. A frequência de pacientes sob violência encaminhados de ambulatórios foi significantemente reduzida em crianças versus adolescentes (27,7 vs. 62%), enquanto encaminhamentos de pronto-socorro foram mais numerosos no primeiro grupo (57,4 vs. 25%, p<0,001). Os tipos de violências e condições pediátricas crônicas foram semelhantes nos dois grupos(p>0,05).

**Conclusões:**

Violência não sexual na nossa população pediátrica foi raramente diagnosticada em um hospital terciário, principalmente negligência, agressão física e psicológica/emocional. Aproximadamente dois terços dos diagnósticos de violência ocorreram em crianças, preferencialmente encaminhadas pelo departamento de emergência. Em contrapartida, cerca de um terço dos diagnósticos de violência ocorreu em adolescentes, encaminhados principalmente pelos ambulatórios.

## INTRODUCTION

Violence against the pediatric population is a serious public health, and human rights and social problem, leading to numerous traumatic consequences that impact families, communities, and nations.^1^ In Brazil, the promotion of children and adolescent health was established in 1990 with the law 8069, named the Child and Adolescents Statute, which ensured to children and adolescents the rights as citizens and made mandatory the violence reporting.^2^

Despite laws and advances in the development of assistance and care strategies, the violence in Brazil is a highly relevant issue. In 2018, data from the Informatics Department of the Unified Health System (DATASUS) showed that violence against children and adolescents occurred in 40% of all cases.^3^ The spectrum of violence in the pediatric population includes mainly physical, psychological/emotional aggression, negligence, sexual, bullying, cyberbullying, and Munchausen by proxy syndrome.^1^ The majority of these studies included pediatric populations under violence situation evaluated predominately at primary and secondary attention.^4,5,6^ Indeed, a recent systematic review, including studies of violence against children and adolescents, carried out in Brazil did not assess patients with pediatric chronic conditions.^4^

Reports involving violence in patients with pediatric chronic diseases followed up in tertiary services were generally restricted to case series of specific pediatric chronic conditions, such as disabilities,^7^ human immunodeficiency virus (HIV),^8,9^ diabetes mellitus,^10,11^ juvenile idiopathic arthritis,^12^ and childhood-onset systemic lupus.^13^

In addition, a recent report with 61 children and adolescents followed up in a tertiary pediatric hospital in Australia showed that patients with chronic medical conditions had a high risk of negligence, particularly when referred by the endocrinology.^14^ However, to the best of our knowledge, there is no study in Latin America, including overall and types of nonsexual violence, hospital referral site, and pediatric subspecialties, especially comparing children and adolescent population under nonsexual violence, who were followed up at a tertiary service.

The objectives of the present study were to assess interpersonal nonsexual violence in children *versus* adolescents and evaluate possible associations of demographic data, type and author of violence, legal referral, laboratorial and imaging examinations, and outcomes in both groups. 

## METHOD

We performed a cross-sectional study, based on a retrospective chart review of 261 children and adolescents under nonsexual violence situation followed up in outpatient and inpatient clinics of our university and tertiary hospital. Patients followed up during 15 consecutive years (January 1, 2005 to June 1, 2020) were recruited. Violence against children and adolescents below 18 years carried out by parents or other caregivers, peers, romantic partners, or strangers were identified using the report at the Violence Multidisciplinary and Multiprofessional Support Pediatric Group of our university hospital. All cases of violence diagnosed in the present service were referred to this multidisciplinary team focused on violence care. A total of 21 subjects were excluded due to incomplete chart data. Therefore, 240 children and adolescents under nonsexual violence situation were systematically evaluated. The Ethics Committee of our hospital approved this study (protocol number 4.040.379). 

The types of interpersonal nonsexual violence against pediatric population, by parents, caregivers, and others at home or at schools studied were physical, psychological/emotional aggression, negligence, bullying, cyberbullying, and Munchausen by proxy syndrome. Physical violence occurs when there is intentional use of force that causes damage.^1^ Neglect occurs when basic needs are not provided.^1^ Emotional or psychological aggression included restricting a child’s or adolescent’s movements, denigration, ridicule, threats and intimidation, discrimination, rejection, and other non-physical forms of hostile behavior. Bullying and cyberbullying were defined as unwanted aggressive behavior by another child/adolescent or group of children/adolescents, who were neither siblings nor in a romantic relationship with the patient, involving repeated physical, psychological, or social harm, generally occurred at schools or digital devices (cell phones, computers, and tablets), respectively.^1^ Munchausen by proxy syndrome was defined as child or adolescent abuse, where parents, caregivers, and other authority figures intentionally created a medical history and induced or fabricated signs or disease in a patient, in order to draw the attention of physicians and other health care professionals.^15,16^

The diagnosis of pediatric chronic condition was established as disease over 3 months of duration, requiring valid methods for diagnosis, standardized tools, or diagnostic classification criteria.^17,18,19^

Data analyses using the electronic data system included demographic data (current age, sex, local of residence, type of residence, the number of rooms in the residences, basic sanitation, and government subsidies), hospital referral site (outpatient clinic, inpatient clinic, and emergency department), the number of medications, type of violence (negligence, physical, sexual abuse, Munchausen syndrome, bullying, and psychological aggression), author of violence (father, mother, and others), legal referral (protective council, children and youth court, and public attorneys), and outcomes (severe injury, impairment of nervous system development, and death). Laboratorial and/or imaging examinations were requested in pediatric patients with physical violence, such as hemoglobin levels, coagulopathy abnormalities, bone fracture, intracranial hemorrhage, and retinal hemorrhage.

We also assessed the following 15 pediatric specialties that followed up patients under violence situation, according to our electronic data system: allergy and immunology, cardiology, endocrinology, gastroenterology, genetics, hematology, hepatology, infectious diseases, nephrology/renal transplantation, neurology, nutrology, oncology, pulmonology, psychiatry, and rheumatology. 

Further analysis divided patients under nonsexual violence situation in two groups according to their current age: children (0–9 years) and adolescents (10–18 years). 

The statistical analyses were performed using the *Statistical Package for the Social Sciences*, version 22, software. The results are presented as median (range) or mean±standard deviation (SD) for continuous variables and number (%) for categorical variables, as recommended. Mann-Whitney U test or Student’s *t*-test compared the continuous variables, as appropriated. For categorical variables, the differences were evaluated by Fisher’s exact test. For all statistical tests, p<0.05 was considered significant. 

## RESULTS

From January 1, 2005 to June 1, 2020, 295,993 children and adolescents attended emergency and outpatient clinics at our university hospital. Of these, nonsexual violence against our pediatric population was diagnosed in 240 (0.1%). Of them, nonsexual violence diagnoses were identified in 148 (61.7%) children and 92 (38.3%) adolescents.

The most common types of violence in 240 children and adolescents were negligence in 156 (65.0%), physical in 62 (25.8%), psychological/emotional aggression in 52 (21.7%), Munchausen by proxy syndrome in 4 (1.7%), and bullying/cyberbullying in 3 (1.3%).

Table 1 presents demographic data, hospital referral site, types of pediatric specialties, pediatric chronic conditions, and the number of current medications in children and adolescents under nonsexual violence situation in a tertiary service experience during 15 years. Regarding the hospital referral site, the frequency of patients under violence referred from outpatient clinics was significantly reduced in children *versus* adolescent (27.7 vs. 62%), whereas the emergency department was higher in the former group (57.4 vs. 25%; p<0.01). Types of pediatric specialties were uniform in both groups. No differences were seen in pediatric chronic conditions between the two groups (49.3 vs. 54.3%, p=0.510) (Table 1). Out of the 123 cases, the most chronic conditions were chronic kidney disease 24 (19.5%), HIV 14 (11.4%), prematurity 9 (7.3%), cerebral palsy 8 (6.5%), and asthma 8 (6.5%).

**Table 1:** Comparison of demographic data, hospital referral site, types of pediatric specialties, pediatric chronic conditions, and the number of current medications between children and adolescents under violence situation in a tertiary service experience during 15 years.

	Children (n=148)	Adolescent (n=92)	p-value
Demographic data			
Female sex	81 (54.7)	51 (55.4)	1.000
Residence in São Paulo state	146 (98.6)	88 (95.7)	0.250
Type of residence			
Rented accommodation	28/73 (38.4)	14/39 (35.9)	0.082
Homeowner	22/73 (30.1)	19/39 (48.7)	
Low-cost housing	23/73 (31.5)	6/39 (15.4)	
Number of rooms in the residence			
≤5	43/44 (97.7)	20/21 (95.2)	0.540
>5	1/44 (2.3)	1/21 (4.8)	
Basic sanitation	19/24 (79.2)	10/11 (90.9)	0.640
Government subsidies	19/81 (23.5)	7/49 (14.3)	0.260
Hospital referral site			
Outpatient clinic	41 (27.7)	57 (62.0)	<0.001
Inpatient clinics	22 (14.9)	12 (13.0)	
Emergency department	85 (57.4)	23 (25.0)	
Types of pediatric specialties			
Hematology and oncology	5 (3.4)	4 (4.3)	NA
Gastroenterology, hepatology, and nutrition	14 (9.5)	4 (4.3)	
Rheumatology, allergy and immunology	3 (2.0)	7 (7.6)	
Nephrology and renal transplantation	20 (13.5)	10 (10.9)	
Neurology and psychiatry	13 (8.8)	9 (9.8)	
Endocrinology	6 (4.0)	7 (7.6)	
Pulmonology	11 (7.4)	4 (4.3)	
Cardiology	2 (1.4)	2 (2.2)	
Infectious disease	8 (5.4)	9 (9.8)	
Pediatric chronic conditions	73 (49.3)	50 (54.3)	0.510
Number of current medications			
≤3	88/124 (71.0)	53/76 (69.7)	0.870
>3	36/124 (29.0)	23/76 (30.3)	

Results are presented as n (%), median (minimum–maximum values), mean±standard deviation. NA: not applicable. Variables with significance statistics in bold (p<0.05).

Table 2 illustrates the type of violence, author of violence, legal referral, examinations, and outcomes in children *versus* adolescents under nonsexual violence situation in a tertiary service experience for 15 years. Types of all violence situations were similar in both groups (p>0.05) (Table 2). Of note, 156 (65.0%) of 240 cases had negligence and 72% of them had pediatric chronic diseases.

**Table 2:** Comparison of type of violence, author of violence, legal referral, examinations, and outcomes between children and adolescents under violence situation in a tertiary service experience during 15 years

	Children (n=148)	Adolescent (n=92)	p-value
Type of violence			
Negligence	95 (64.2)	61 (66.3)	0.780
Physical	41 (27.7)	21/91 (23.0)	0.450
Munchausen syndrome	3 (2.0)	1 (1.0)	1.000
Bullying/cyberbullying	0 (0)	3 (3.3)	–
Psychological/emotional aggression	27 (18.2)	25 (27.2)	0.110
Author of violence			
Father	54/142 (38.0)	29/91 (31.9)	0.510
Mother	20/142 (14.1)	17/91 (18.7)	
Others	68/142 (47.9)	45/91 (49.4)	
Legal referral			
Protective Council	100 (67.6)	57 (61.9)	0.400
Child and Youth Court	70 (47.3)	45 (48.9)	0.890
Public Attorneys	3 (2.0)	0	–
Laboratorial and imaging examinations			
Hemoglobin, g/dL (n=67)	11.5±2.3*	10.9±3.5**	0.500
Coagulopathy abnormalities	0/10	1/2 (50)	–
Bone fracture	5/14 (35.7)	0/4	–
Intracranial hemorrhage	8/16 (50.0)	1/5 (20.0)	0.340
Retinal hemorrhage	0/6	0/6	–
Outcomes			
Severe injury			
Impairment of nervous system development	2 (1.4)	1 (1.1)	1.000
Death	1 (0.7)	0 (0)	–

Results are presented as n (%), median (minimum–maximum values), mean±standard deviation. STI: sexually transmitted infectious. *n=45, **n=22.

Three patients under nonsexual violence situation had severe injury (n=3/240 [1.2%]) with progressive impairment of nervous system development. Two infants had shaken baby syndrome with cognitive–behavioral deficit and without retinal hemorrhages. One female adolescent had chronic neuropathy requiring tracheostomy after severe physical and domestic violence. 

Other female patient had previous cerebral palsy, requiring gastrostomy, ventricular-peritoneal shunt valve, and anticonvulsants (phenobarbital and clonazepam). At 4 years, she was hospitalized due to gastrostomy bleeding and dehydration, aggravated by negligence. After 1 week, she died due to complications of septic shock.

## DISCUSSION

Our findings show that nonsexual violence against our pediatric population was rarely diagnosed in a tertiary hospital, although there were some occurrences, mainly negligence, physical, and psychological/emotional aggression.

Approximately two-third of nonsexual violence diagnosis occurred in children referred by the emergency department. In contrast, approximately one-third of nonsexual violence diagnosis occurred in adolescent referred by outpatient clinics. 

The strengths of the present study are the inclusion of patients followed up for a long period in a university service of Latin American, with a half of them suffering from pediatric chronic conditions followed by different pediatric specialties.^17^ In addition, our tertiary center has also a Violence Multidisciplinary and Multiprofessional Support Pediatric Group. The main purposes of this relevant support group are early diagnosis of violence in all outpatient and inpatient clinics; minimizing the violence outcomes; general and specific treatments for children and adolescents under violence situation; mental health support for the aggressor, victim, and family; and legal referral support. 

We found that negligence was the most important type of violence, observed in two-thirds of our patients, and 72% of them have pediatric chronic conditions, as also reported by other authors.^20^ The high frequency of neglect violence situation in both children and adolescents groups with chronic illnesses may be related to the use of various medications, high frequency of multidisciplinary team appointments, several clinical and surgical procedures, cognitive dysfunction, and mobility restrictions that may impact caregivers.^14^ In addition, a recent meta-analysis study showed that physical and/or behavioral mental issue was an independent risk factor for negligence.^21^

One-fourth of children and adolescents were victims of physical violence herein. This finding was observed by other reports, from 7.9 to 44.7%, especially among young subjects.^2,22^ Children are predominantly victims of domestic violence, and their mothers were the main aggressor.^22^ Although adolescents are generally victims of community violence.^23^

Approximately two-third of nonsexual violence diagnosis occurred in children referred by the emergency department. In fact, our patients under physical violence were initially assessed in the emergency department to exclude organ and system damage, such as bone fracture, intracranial hemorrhage, and retinal hemorrhage, as observed in the present study. 

Interestingly, adolescents under nonsexual violence diagnoses were referred predominately by rheumatology and allergy and immunology specialties. In fact, the majority of patients followed up by these specialties are adolescents with complex diseases, requiring multiples appointments and various immunosuppressive and immune-mediated therapies.^17,18,19^

Furthermore, psychiatry’s referral was observed only in adolescents with nonsexual violence diagnosis. Indeed, mental health impact may be related to violence in adolescents’ populations, particularly in those with previous psychiatric conditions. A systematic review and meta-analysis study showed a causal relationship between nonsexual pediatric maltreatment and a range of mental disorders, drug use, suicide attempts, sexually transmitted infections, and risky sexual behavior.^24^

Importantly, all of our patients under violence were promptly referred to the responsible legal sectors by social workers, mainly to the Protective Council and Child and Youth Court. Thus, interdisciplinarity with all social sectors is relevant to address violence in pediatric populations. This fact reinforces the culture of vigilance against all types of violence situation for pediatric patients, involving all professionals, pediatric subspecialties, and different locations of the hospital.

The main limitation of the present study is its cross-sectional nature, based on retrospective chart review. In addition, we excluded the important analysis of sexual violence, since this abuse is not followed in our pediatric hospital. Patients are immediately referred to another division of the same tertiary and academic hospital.

Nonsexual violence against our pediatric population was rarely diagnosed in a tertiary hospital, mainly negligence, physical, and psychological/emotional aggression. Approximately two-third of violence diagnosis occurred in children, referred mainly by the emergency department. In contrast, approximately one-third of violence diagnosis occurred in adolescents, referred mostly by outpatient clinics.

## References

[1] World Health Organization. (2020). Violence against children [homepage on the Internet]..

[2] Santos TM, Pitangui AC, Bendo CB, Paiva SM, Cardoso MD, Melo JP (2017). Factors associated with the type of violence perpetrated against adolescents in the state of Pernambuco, Brazil. Child Abuse Negl..

[3] Brazil - Ministério da Saúde (2020). Banco de dados do Sistema Único de Saúde..

[4] Macedo DM, Foschiera LN, Bordini TC, Habigzang LF, Koller SH (2019). Systematic review of studies on reports of violence against children and adolescents in Brazil. Cienc Saude Colet..

[5] Hillis S, Mercy J, Amobi A, Kress H (2016). Global prevalence of past-year violence against children: a systematic review and minimum estimates. Pediatrics.

[6]  Corboz J, Hemat O, Siddiq W, Jewkes R (2018). Children’s peer violence perpetration and victimization: prevalence and associated factors among school children in Afghanistan. PLoS One.

[7]  Jones L, Bellis MA, Wood S, Hughes K, McCoy E, Eckley L (2012). Prevalence and risk of violence against children with disabilities: a systematic review and meta-analysis of observational studies. Lancet.

[8]  Skeen S, Macedo A, Tomlinson M, Hensels IS, Sherr L (2016). Exposure to violence and psychological well-being over time in children affected by HIV/AIDS in South Africa and Malawi. AIDS Care..

[9] Cluver L, Meinck F, Toska E, Orkin FM, Hodes R, Sherr L (2018). Multitype violence exposures and adolescent antiretroviral nonadherence in South Africa. AIDS..

[10] Andrade CJ, Alves CA (2019). Relationship between bullying and type 1 diabetes mellitus in children and adolescents: a systematic review. J Pediatr (Rio J)..

[11] Iqbal AM, Kumar S, Hansen J, Heyrman M, Spee R, Lteif A (2020). Association of adverse childhood experiences with glycemic control and lipids in children with type 1 diabetes. Children (Basel)..

[12] van Weelden M, Lourenço B, Viola GR, Aikawa NE, Queiroz LB, Silva CA (2016). Substance use and sexual function in juvenile idiopathic arthritis. Rev Bras Reumatol Engl Ed..

[13] van Weelden M, Queiroz LB, Lourenço DM, Kozu K, Lourenço B, Silva CA (2016). Alcohol, smoking and illicit drug use in pediatric systemic lupus erythematosus patients. Rev Bras Reumatol Engl Ed..

[14] Parmeter J, Tzioumi D, Woolfenden S (2018). Medical neglect at a tertiary paediatric hospital. Child Abuse Negl..

[15] Sousa Filho D, Kanomata EY, Feldman RJ, Maluf A (2017). Munchausen syndrome and Munchausen syndrome by proxy: a narrative review. Einstein (Sao Paulo)..

[16] Kuhne AC, Pitta AC, Galassi SC, Gonçalves AM, Cardoso AC, Paz JA (2019). Munchausen by proxy syndrome mimicking childhood-onset systemiclupus erythematosus. Lupus..

[17] Alveno RA, Miranda CV, Passone CG, Waetge AR, Hojo ES, Farhat SC (2018). Pediatric chronic patients at outpatient clinics: a study in a Latin American University Hospital. J Pediatr (Rio J)..

[18] Passone CG, Grisi SJ, Farhat SC, Manna TD, Pastorino AC, Alveno RA (2019). Complexity of pediatric chronic disease: cross-sectional study with 16,237patients followed by multiple medical specialties. Rev Paul Pediatr..

[19] Ramos GF, Ribeiro VP, Mercadante MP, Ribeiro MP, Delgado AF, Farhat SCL (2019). Mortality in adolescents and young adults with chronic diseases during 16 years: a study in a Latin American tertiary hospital. J Pediatr (Rio J)..

[20] Flaherty EG, Stirling J (2010). American Academy of Pediatrics. Committee on Child Abuse and Neglect. Clinical report — the pediatrician’s role in child maltreatment prevention. Pediatrics..

[21] Mulder TM, Kuiper KC, Put CE, Stams GJ, Assink M (2018). Risk factors for child neglect: A meta-analytic review. Child Abuse Negl..

[22] Franzin LC, Olandovski M, Vettorazzi ML, Werneck RI, Moysés SJ, Kusma SZ (2014). Child and adolescent abuse and neglect in the city of Curitiba, Brazil. Child Abuse Negl..

[23] Reichenheim ME, Souza ER, Moraes CL, Mello Jorge MH, Silva CM, Souza Minayo MC (2011). Violence and injuries in Brazil: the effect, progress made, and challenges ahead. Lancet..

[24] Norman RE, Byambaa M, De R, Butchart A, Scott J, Vos T (2012). The long-term health consequences of child physical abuse, emotional abuse, and neglect: a systematic review and meta-analysis. PLoS Med..

